# Structure and Grammaticalization of Serial Verb Constructions in Sign Language of the Netherlands—A Corpus-Based Study

**DOI:** 10.3389/fpsyg.2018.00993

**Published:** 2018-07-17

**Authors:** Sascha Couvee, Roland Pfau

**Affiliations:** Department of Linguistics, University of Amsterdam, Amsterdam, Netherlands

**Keywords:** serial verb construction, Sign Language of the Netherlands, grammaticalization, corpus, agreement

## Abstract

In serial verb constructions (SVCs), multiple independent lexical verbs are combined in a mono-clausal construction. SVCs express a range of grammatical meanings and are attested in numerous spoken languages all around the world. Yet, to date only few studies have investigated the existence and functions of SVCs in sign languages. For the most part, these studies—including a previous study on Sign Language of the Netherlands (NGT)—relied on elicited data. In this article, we offer a cross-modal typological contribution to the study of SVCs by investigating the phenomenon based on naturalistic corpus data from NGT. A search of the Corpus NGT yielded 41 mono-clausal utterances in which one of a closed set of verbs—namely go, give, take, and call—combines with another lexical verb. While the combinations we found are in important respects reminiscent of SVCs described for spoken languages, our data also confirm the previous finding that the fixed verb in the SVC serves to express agreement (by means of spatial modulation) when the other verb cannot do so. In addition, we identified some novel uses of the verbs go and give: (i) go functioning as a future tense marker and (ii) give functioning as a light verb. We will also discuss aspects of the grammaticalization of SVCs in NGT: from lexical verb to light verb to auxiliary, again offering some comparison to grammaticalization paths described for spoken languages.

## Introduction

Verbs and verbal inflection in sign languages have received considerable attention since the early days of sign language linguistics (Fischer and Gough, 1978; Klima and Bellugi, 1979; Padden, 1983), and their properties and appropriate characterization are still under scrutiny in recent studies (Meir, 2002; Lillo-Martin and Meier, 2011; Wilbur, 2013; Schembri et al., 2016). One of the issues that is hotly debated in the literature is the applicability of certain morphosyntactic notions that have been established on the basis of spoken languages to the domain of sign languages, that is, the cross-modal applicability of these notions. Obviously, given that sign languages make use of different articulators—not only the hands, but also the head, face and body—in the transmission of lexical and grammatical information, it cannot be taken for granted that concepts and models developed for spoken languages can be applied to languages in the visual-gestural modality. Yet, after 50 years of research, there is now broad consensus that many aspects of the structural make-up of sign languages are modality-independent; e.g., a prosodic hierarchy, relativization strategies, and reduplication as a morphological process—to give just three examples from different domains (cf. Wilbur, 2009; Sandler, 2012; Pfau and Steinbach, 2016). At the same time, however, there is also growing awareness that the modality of signal transmission impacts certain aspects of the lexicon and grammar (Meier, 2012). Think, for instance, of the availability of two identical articulators, the two hands, and the use of the signing space in front of the signer's body for grammatical purposes. Yet, despite the modality-specific flavor of these articulatory strategies, some sign linguists have argued that, at a sufficiently abstract level, they can be modeled in a modality-independent way (e.g., Pfau et al., submitted; Kimmelman, 2017).

Verbs and spatial grammar also figure prominently in this paper, which focuses on a specific type of verbal construction: serial verb constructions (SVCs) in Sign Language of the Netherlands (*Nederlandse Gebarentaal*—NGT). SVCs are mono-clausal constructions which contain multiple lexical verbs. They are thus clearly distinct from (i) complex constructions such as e.g., coordination (*She came and danced*) and (ii) auxiliary-verb constructions (*They must leave*). Across spoken languages, SVCs have been found to express a range of grammatical meanings, including a sequence of actions or a cause and effect relation between the actions expressed by the verbs (Aikhenvald, 2006).

Although there is an extensive body of literature on SVCs in spoken languages, there has been comparably little research on SVCs in sign languages. Recently, however, a 1996 presentation on SVCs in NGT, which had been circulated as a manuscript, has been published as a *Paper from the Sign Language Underground* (Bos, 1996/2016). The current study is inspired by Bos' seminal work and continues the investigation of SVCs in NGT. However, while Bos' research was based on elicited data, we investigate the occurrence of this construction type in naturalistic corpus data. To that end, we searched the Corpus NGT, a large online corpus with videos of conversations between signers, for utterances which contain one of the verbs that Bos had previously identified (go, give, take, and call) in combination with another lexical verb. Just like Bos, we analyze morphosyntactic and syntactic properties of these constructions: the distribution of agreement inflection, word order, and argument structure. Furthermore, we add to the picture some speculations on new uses of some of the verbs participating in SVCs: (i) give functioning as a light verb and (ii) go functioning as a future tense marker. Our findings contribute to the existing knowledge on SVCs and indicate that more research on this topic in more sign languages will likely reveal striking similarities to SVCs found in spoken languages as well as potentially modality-specific properties.

We start our investigation in section Serial Verb Constructions Across Language Modalities by offering an overview of previous research on SVCs in spoken and sign languages, including also a summary of the findings of Bos (1996/2016). In section Methodology, we describe our methodology, in particular, the application of criteria for identifying SVCs. Results per verb are presented in section Results, where we also comment on the range of free verbs that the fixed verbs combine with. In section A Cross-Linguistic Perspective on Serial Verb Constructions, we then discuss structural and functional properties of SVCs from a cross-linguistic perspective, and we compare the patterns to those reported by Bos. Speculations about possible grammaticalization paths are offered in section Grammaticalization. Section Conclusion concludes the paper.

## Serial verb constructions across language modalities

Obviously, multiple verbs are commonly combined within a sentence—think, for instance, of auxiliary-verb constructions (e.g., *They have eaten* or *You should pay*) and infinitival complements (e.g., *She came to dance*). However, not all of these combinations qualify as SVCs. In fact, most of them don't. In section Defining Properties of Serial Verb Constructions, we will introduce the defining properties of SVCs, extracted for the most part from typological overviews by Aikhenvald (2006) and Haspelmath (2016), which are based entirely on data from spoken languages. We then turn to sign languages. We summarize findings from various sign languages, including NGT, in section Serial Verb Constructions in Sign Languages before highlighting the contribution of the present study in section Contribution of the Present Study.

### Defining properties of serial verb constructions

Based on a wealth of data from typologically diverse spoken languages, researchers have identified a number of general defining properties of SVCs. According to Haspelmath (2016, p. 296), a SVCs “is a mono-clausal construction consisting of multiple independent verbs with no element linking them and with no predicate-argument relation between the verbs.” SVCs have been identified in many different spoken languages from all around the world, such as Oceanic languages, Amazonian languages, the languages spoken in New Guinea, Southeast Asian languages, West African languages, and creole languages (Aikhenvald, 2006, p. 52). In the following, we will flesh out the five properties (or “key components”) that make up Haspelmath's definition, also adding to the picture criteria offered by Aikhenvald (2006).

#### (i) Independent verbs

The verbs that participate in SVCs are independent verbs, that is, forms that can express a dynamic event in a non-elliptical utterance without any other verb. This is true for the verbs involved in the Cantonese SVC in (1) and the Saramaccan SVC in (2). 
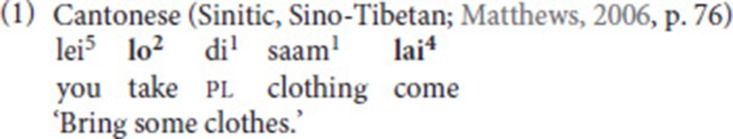
 
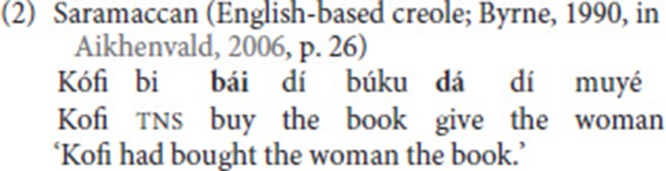


Furthermore, it has been observed, that cross-linguistically, some verbs tend to occur more frequently in SVCs than other verbs. Verbs that are commonly used in SVCs are basic verbs of motion [e.g., “come” and “go”; cf. (1)], direction, posture (e.g., “sit” and “stand”), and location (Aikhenvald, 2006). Some languages also employ valency-increasing or argument-adding SVCs. A verb often used in this construction type is “give,” which can also add a benefactive meaning, as is illustrated by the example from Saramaccan in (2).

#### (ii) Monoclausality

Crucially, the two (or more) verbs belong to a single clause. Consequently, SVCs are also conceptualized as a single event. In the Cantonese example in (1), for instance, there is no taking-event that would be independent from the coming-event (e.g., take some clothes from a pile of clothes and then come). Rather, the combination of the two verbs within a single clause yields the meaning “bring.” The Taba example in (3) is slightly different, as the dying-event is a result of the biting-event. Still, given the causal relationship, that is, the fact that the death is a direct consequence of the biting, we are dealing with a single event. It is, for example, impossible that the pig died for another reason than the bite, or that it only died from the bite 2 days later. All three examples provided so far also illustrate that the verbs in an SVC do not have to be adjacent. 
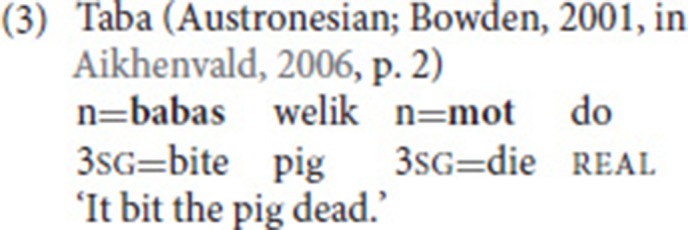


Monoclausality of the SVC is further evidenced by the fact that all participating verbs must share tense, aspect, and polarity specification. This implies, for instance, that it is impossible to negate just one of the verbs [e.g., taking the clothes and not coming in (1)]. Yet another important indication that SVCs are monoclausal is that there are no pauses or intonational breaks within the construction; that is, the construction has the same intonational properties as a monoverbal clause (Aikhenvald, 2006).

#### (iii) No linking elements

Related to the monoclausality constraint is the fact that there should be no coordinating or subordinating element between the verbs. This constraint excludes English multi-clausal structures like *She came and danced*. Still, English, a non-serializing language, allows for the double verb construction in (4a), which, on the surface, resembles a SVC (Bjorkman, 2016, p. 54). 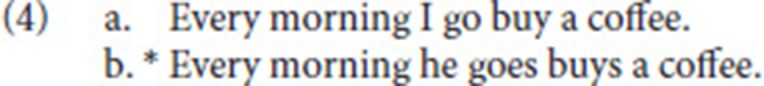


However, constructions of this type are not considered SVCs since they are not productive: they can only be used in a specific context and only a few verbs are allowed. Overt marking of the verbs as in (4b) results in ungrammaticality. Additionally, it is possible to add a conjunction between the verbs, without changing the meaning (Aikhenvald, 2006).

In serializing languages, minimally different multi-clausal structures commonly exist next to SVCs. Compare the Taba example in (5) to the one provided in (3) above. The example does not include a coordinate conjunction but use of the causative marker and the object pronoun indicates that we are dealing with two clauses [i.e., the argument *pig* is not shared—see point (iv)]. 
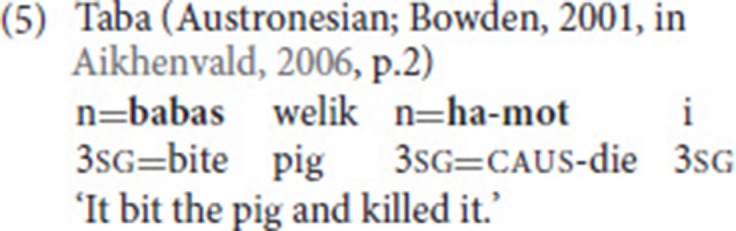


#### (iv) Argument structure properties

Some observations can be made concerning the argument structure of the verbs involved in the SVC. First, the verbs can have different transitivity values. Examples (1) and (3), for instance, involve an intransitive and a transitive verb, while (2) includes a transitive and a ditransitive verb. Second, the verbs in an SVC commonly share arguments (Aikhenvald, 2006). In (1), the second person pronoun is the subject of both verbs (*take* and *come*); in (3), *pig* is the object of the first verb (*bite*), but the subject of the second one (*die*).

Third, as for argument structure properties, Haspelmath (2016) adds yet another criterion: One of the verbs should not be (part of) an argument of the other verb. This criterion excludes complement clause constructions like the one from Samoan in (6), where *swim* is part of an infinitival complement embedded under *know* (the same criterion excludes English examples like *He helped me wash the car*). 
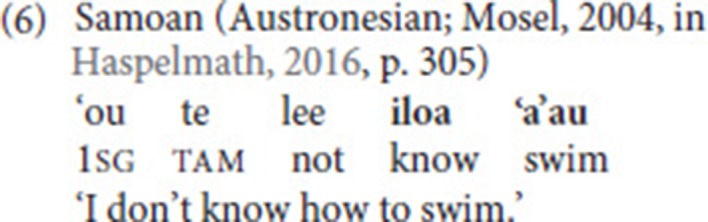


#### (v) Construction

Finally, Haspelmath (2016) states that a SVC must be a productive, schematic construction with a concrete meaning. The meaning of the SVC must be determinable from its elements. In other words, constructions that involve non-compositional combinations of verbs do not fall under the definition of SVC, as such idiomatic constructions are usually not productive [as is true e.g., for (4)]. In this context, Haspelmath (2016, p. 297) remarks: “In very general terms, typology does not take into account idiomatic expressions, and confines itself to regular patterns in languages”^1^.

Furthermore, constructions that comply with these five criteria can be divided into two broad classes: asymmetrical SVCs and symmetrical SVCs. In asymmetrical SVCs, one of the verbs is from a restricted semantic class while the other verb is taken from a non-restricted class. The “free” verb describes the event while the restricted verb adds to the construction a specification, such as direction or manner, or tense-aspect meaning. In (2), for instance, *bái* (“buy”) is the free verb and *dá* (“give”) is the restricted verb providing directional meaning. The fixed verbs in asymmetrical SVCs tend to grammaticalize, as evidenced by semantic bleaching and (at times) phonological erosion, often at the same time preserving their status as independent lexical verbs (Lord, 1993). In contrast, the verbs in symmetrical SVCs are all taken from a semantically unrestricted class. According to Aikhenvald (2006, p. 28), the order of verbs in symmetrical SVCs is iconic, that is, “it follows the temporal sequence of the subevents,” as is evident from example (3).

### Serial verb constructions in sign languages

As mentioned previously, to date, SVCs have not received a lot of attention in the sign language literature. An early study on American Sign Language (ASL) focused on one specific construction type, namely “serial verbs of motion” which—as the name implies—express a motion event. As we will include in our discussion SVCs with motion verbs, we will briefly address this study, as well as a subsequent study on the phenomenon, in section Serial Verbs of Motion. In section Serial Verb Constructions in NGT, we then summarize the findings of Bos (1996/2016), which constitute the starting point for our corpus-based investigation. However, before turning to these previous studies, we introduce in section Space and Prosody in Sign Languages aspects of sign language grammar that will play a role in our discussion of sign language SVCs.

#### Space and prosody in sign languages

Two of the components of sign language grammar we wish to briefly introduce pertain to the modification of predicates, namely spatial agreement and classifiers; the third one concerns the marking of prosodic structure.

In most sign languages studied to date, a subgroup of verbs, the so-called “agreeing” or “directional” verbs, can be spatially modified to mark the subject and/or object (e.g., in NGT give, visit, help). Typically, in these verbs, the movement starts at the locus in signing space associated with the subject and proceeds toward the locus associated with the object. These can be loci of present referents (e.g., signer or addressee) or arbitrary loci that have been introduced for non-present referents by means of a pointing sign. An important qualification is that not all verbs can be modified in this way (Padden, 1983; Meir, 2002). Body-anchored verbs, like e.g. love and understand in NGT, do not allow spatial modification—such verbs are referred to as “plain” verbs. Different sign languages employ different strategies for marking argument structure with plain verbs, e.g., word order or the use of a dedicated auxiliary (see section Grammaticalization for further discussion). Moreover, in some verbs, the handshape can be modified to represent certain shape characteristics of a referent, be it a subject or an object (e.g., in NGT give, be-located, move)—such meaningful handshapes are commonly referred to as “classifiers” and the verbs they combine with as “classifier predicates.”

In (7a), we present an NGT example that illustrates both types of modification. First, the non-present referent colleague is localized at locus 3a by means of the pointing sign index; subsequently, the verb give moves from this locus toward the signer, thus expressing the meaning “he gives me.” At the same time, the handshape is changed to a T-handshape (thumb and index finger make contact, other fingers extended), a handling (hd) classifier representing the handling of a long thin object (other classifier types will be introduced in the next section).^2^ 
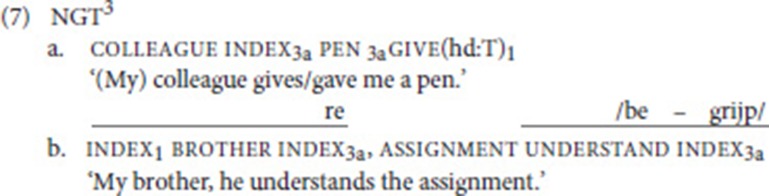


Prosodic constituency in sign languages is marked by manual and non-manual signals (e.g., Nespor and Sandler (1999), Dachkovsky and Sandler (2009); see Sandler (2012) for overview). In (7b), the topic constitutes an intonational phrase (IP). Manually, this is marked by a phrase-final hold and a pause. As for non-manual marking, we observe raised eyebrows (re) accompanying the topic; in addition, the right boundary of an IP is commonly marked by an eye blink. Of importance in our context is the fact that mouthings, i.e., silent articulations of a (part of a) Dutch word corresponding to the meaning of a sign, can also serve a prosodic function, as they may spread to mark a prosodic word (Sandler, 1999; Crasborn et al., 2008a)^4^. This is also observed in (7b), where the mouthing accompanying the verb understand (Dutch *begrijpen*) spreads onto the sentence-final subject pronoun. The first syllable of the Dutch stem is articulated while the verb is signed, the second syllable while the hand moves toward locus 3a; in addition, the movement of the verb and the pronoun are fused into one continuous contour. In combination, mouthing and movement indicate that the pronoun is cliticized to the verb.

#### Serial verbs of motion

Supalla (1990) describes a construction type in ASL which refers to a motion event and systematically includes two verbs, one expressing the movement path and the other the manner of movement; he labels this construction “serial verbs of motion.” A representative example is given in (8). After introducing the moving entity, the signer first expresses the manner of motion, namely running, by using a classifier construction (Supalla, 1986). Both hands have a slightly bent 1-handshape (index finger extended) with the fingertips pointing downward; this is a body part (bp) classifier referring to the legs. It is the combination of these handshapes with the alternating movement of the two hands that yields the meaning of running. Supalla (1990, p. 133) stresses the fact that “the hands must remain in place, in front of the signer's body, while these movements are made.” The path is only expressed subsequently by a one-handed sign. Once again, we observe a 1-handshape, but this time with the fingertip oriented upwards. This is a whole entity (we) classifier referring to the person as a whole. The lexical meaning of the second verb is “go,” and it expresses the path by means of its movement properties, namely moving upward in a zigzag motion. In other words, (8) contains two lexical verbs which join forces to express the complex movement^5^. 
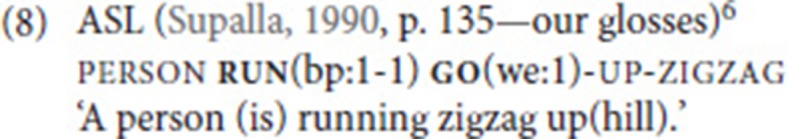


Supalla argues that a multi-verb construction is required in this case, as ASL has a grammatical restriction that bans the simultaneous combination of a body part classifier with a sign expressing path. Hence, the two meaning components have to be expressed independently. He further identifies the following two grammatical restrictions: (i) the manner verb always precedes the path verb, and (ii) no other constituent can intervene between the two verbs of a motion SVC—in crucial contrast to what we observed in the spoken language examples in section Defining Properties of Serial Verb Constructions.

Taking Supalla's seminal work as a starting point, Benedicto et al. (2008) compare syntactic properties of motion SVCs in ASL, Catalan Sign Language (LSC), and Argentinean Sign Language (LSA). They find that the constraint on order also holds for LSA, but not for LSC, where both orders are grammatical. The order path–manner is illustrated in example (9a). Furthermore, they observe a sequence that they refer to as “sandwich” pattern, in which one of the verbs is doubled (Fischer and Janis, 1990). Both LSC and LSA allow for the order manner–path–manner, and LSC in addition displays the order path–manner–path. The former sandwich pattern is illustrated by the LSA example in (9b). As in ASL, in both LSC and LSA, no constituent can intervene between the two verbs. 
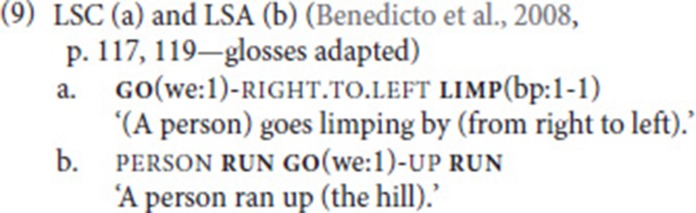


Costello (2016) argues that these constructions, despite the fact that they include verbs of motion, are not SVCs of the directional type (comparable to (1) above). Rather, “it is the manner verb that categorizes this type of construction, making it a symmetrical manner construction” (Costello, 2016, p. 257), comparable to the Ewe example in (10). 
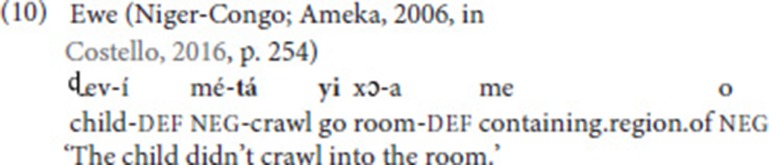


While we agree with the classification as a manner, rather than a directional, SVC for the above cases, we have to keep in mind that the verb “go” is usually considered a member of the restricted class of verbs. We thus think that we are actually dealing with asymmetrical SVCs in which the free verb (*run, limp, crawl*) describes the event while the restricted verb adds specification of direction.

Motion SVCs of this type are relevant to our study, as we, following Bos (1996/2016), will also include SVCs with the motion verb go. However, as will become evident in section SVCs With Movement Verb go, the motion SVCs in our dataset are different, as the verb go is indeed fixed, that is, it is a general directional verb and not a complex predicate that combines with classifier or path morphemes.

#### Serial verb constructions in NGT

NGT is a sign language used in the Netherlands which has developed over the last 200 years (Tijsseling, 2014). Aspects of the grammar of NGT are fairly well described, at least compared to many other sign languages^7^. The basic word order is usually claimed to be SOV (Koolhof and Schermer, 2009; Brunelli, 2011), but recent corpus studies find that SVO is also commonly used (Oomen and Pfau, 2017). Bos (1996/2016) is the only previous work on SVCs in NGT^8^. Interestingly, throughout the article, Bos refers to the constructions under investigation as “double verb constructions”; only in the conclusion, she points out that the double verb constructions have almost all the characteristics of SVCs in spoken languages—and the title of her paper reflects this conclusion.

For her study, Bos collected data from nine deaf participants (age 21–27), who were active members of the Deaf community and used NGT on a regular basis with deaf family and/or friends. The participants were filmed while performing different tasks which had been designed to elicit the use of 80 NGT verbs. The recordings were transcribed, and certain characteristics of the signers' productions [e.g., verb(s) used, expression and locus of agreement, properties of the arguments] were entered into a database. Subsequent analysis yielded 116 double verb constructions.

One of the verbs in these constructions always comes from a fixed set of four verbs: go, give, take, or call; the constructions therefore are candidates for asymmetrical SVCs. All four verbs have fairly general semantics and are also commonly found in SVCs in spoken languages (Aikhenvald, 2006). In contrast, the other verb in the NGT double verb constructions, the free verb, comes from a larger group of verbs. Yet, Bos observes that the free verbs are always from the same semantic class as the fixed verbs. The verb go combines with other verbs of movement such as walk (11a). The transfer verbs give and take combine with free verbs that also express the transfer of an object such as borrow or pay (11b). Finally, call tends to co-occur with other speech act verbs, e.g., ask (11c). As for the order of the two verbs, Bos observes that the free verb usually precedes the fixed verb. 
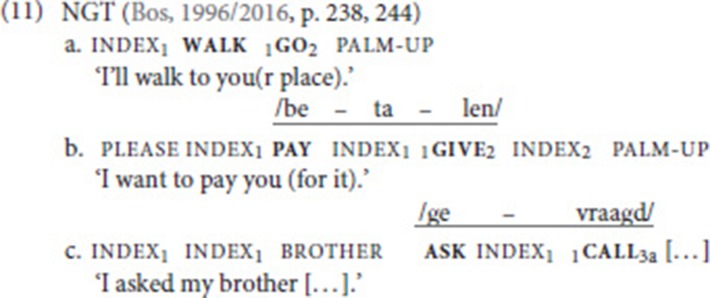


Bos (1996/2016) offers several arguments for analyzing these (and similar) constructions as SVCs. First, she stresses that the double verb constructions are mono-clausal, as they denote a single event. That is, a translation in which the verbs are not in the same clause, e.g., “I want to pay and give you” for (11b), does not capture the meaning of these sentences. A mono-clausal analysis is further supported by the observation that it is not possible to negate only part of the construction, i.e., one of the verbs. In (11a), for instance, a simultaneous negative headshake could not be used to express that I am going to you but not by means of walking (cf. the Ewe example in (10), which is negated by the discontinuous morpheme *mé…o*). Second, the verbs in the double verb constructions share arguments, as is also commonly the case in SVCs found in spoken languages.

Third, the examples that Bos analyzed also display prosodic characteristics of a single clause. Crucially, there are never intervening pauses in between the verbs; the verbs are under one fluent intonation contour. In examples (11bc), the mouthing lends further support to this claim. Despite the presence of an intervening subject pronoun, in both examples, a mouthing associated with the first verb spreads over the second verb (*betalen* is the infinitival form of “pay,” *gevraagd* is the participle of “ask”), thus defining a prosodic word, which again indicates that these are mono-clausal constructions.

In a sense, the fixed verb does not contribute much to the meaning of the SVC as a whole. Consequently, the translations in (11) only include the meaning of the free verb. One may therefore wonder why an SVC is used in the first place. Bos (1996/2016) argues that the main function of the fixed verb is to express agreement whenever the free verb is a plain verb that cannot be spatially modified. Note that in all three examples in (11), the fixed verb carries subscripts which indicate that the verb is spatially modified. The free verbs walk, pay, and ask are not modified in this way. We will discuss this interesting observation in more detail in section Function of SVCs. For now, suffice it to say that Bos (1996/2016) concludes that the double verb constructions she encountered in the elicited data share interesting properties with asymmetrical SVCs in spoken languages^9^.

### Contribution of the present study

Equipped with background knowledge on SVCs in spoken and sign languages, we can now proceed to our study. As mentioned in the previous section, the seminal investigation by Bos (1996/2016) focused on SVCs in elicited utterances. In contrast, we examine the occurrence of SVCs in naturalistic language data. We extracted instances of SVCs from the Corpus NGT (Crasborn and Zwitserlood, 2008; Crasborn et al., 2008b), a database which consists of signed conversations between two signers. Adding corpus data to the picture is a worthwhile endeavor, as recent corpus-based studies on NGT and other sign languages have shown that generalizations that have been established based on elicited data and/or grammaticality judgments sometimes have to be reconsidered once corpus data are added to the data pool. This does not necessarily mean that these generalizations are wrong, but it may indicate that they are too strict, as the corpus data reveal more variation than has previously been assumed. For NGT, such variation has been identified in the domains of word order, including the position of manual negation (Oomen and Pfau, 2017), agreement (Legeland, 2016), and the use of mouthings (Bank, 2014)^10^. An important contribution of the present study is thus testing Bos' generalizations on the basis of data from the Corpus NGT.

In addition, we will pay attention to syntactic properties of the SVCs, such as order of the verbs and the nature of intervening elements; these properties are mentioned in Bos (1996/2016), but are not discussed in detail. Finally, some examples extracted from the corpus data will allow us to offer some speculations on grammaticalization.

## Methodology

Data were extracted from the Corpus NGT (Crasborn and Zwitserlood, 2008; Crasborn et al., 2008b), a large, partly annotated corpus including videos of 92 signers (recorded 2006–2008). The data consist of free and elicited conversations between pairs of signers from different regions in the Netherlands. Most of the corpus is freely available and annotated in Dutch; part of it is translated into Dutch and English.

### Participants

For our study, we used data from 35 signers (15 female), aged 18–84. The participants in this sample came from different regions in the Netherlands: Groningen (17), Amsterdam (11), St.-Michielsgestel (4), Voorburg (2), and Rotterdam (1). All of them were deaf from birth or became deaf soon after birth and started using NGT at an early age (before the age of 4).

### Procedure: searching the corpus

The Corpus NGT is partly annotated in ELAN. ELAN (Crasborn and Sloetjes, 2008) allows for searches in the annotations. Our first step was to search for all instances of the four verbs discussed by Bos (1996/2016), namely, go (Dutch *gaan*), give (*geven*), take (*pakken*), and call (*roepen*); to this, we added the verb come (*komen*). With respect to this latter verb, a few explanations are in place. As pointed out in section Defining Properties of Serial Verb Constructions, the verb meaning “come” commonly participates in SVCs. More importantly, however, the verbs that are glossed as go and come in the Corpus NGT have the same phonological form: the index finger is extended and slightly bent, and the hand performs a path movement in the signing space. What motivates use of the one gloss over the other is the direction of movement, which is away from the signer for go, but toward the signer for come (e.g., “I go to her” vs. “She comes to me”). It seems to us that the use of two different glosses does not reflect a difference in meaning; rather it is based on the spoken language. Therefore, in the remainder of this paper, we will collapse go and come, and gloss them consistently as go. In other words: some of our glossed examples differ from the annotations in this respect.

Based on corpus data, we first confirmed that all five verbs can be used independently (criterion (i) in section Defining Properties of Serial Verb Constructions). That this is indeed the case, is illustrated by the examples in (12) (see section Procedure: Applying the SVC criteria for explanation of the example codes).^11^ 
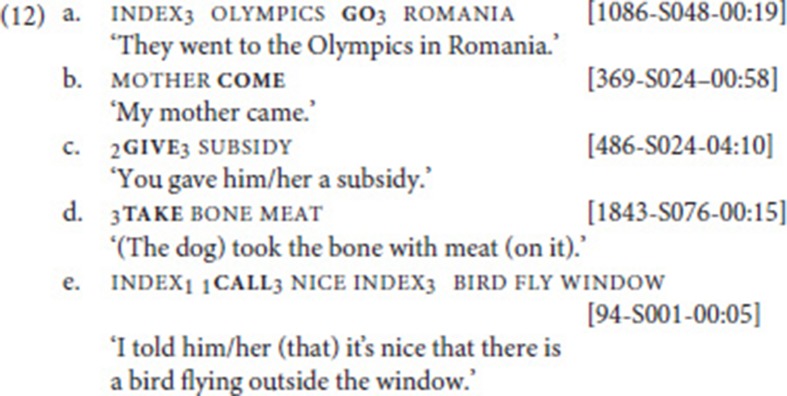


Once the independent use of these verbs had been established, we started our search for SVCs. Given that the corpus is not annotated for word class, it was not possible to easily search for one of the five verbs in combination with another verb. Therefore, all instances had to be reviewed manually.

Our initial search yielded a number of irrelevant occurrences, in particular, glosses which included the search item as part of another verb with a more specific meaning, for instance, weg**gaan** (“to leave”; literally “go away”) or les**geven** (“to teach”; literally “give lesson”). Once we excluded these occurrences, 1,389 cases remained for further analysis. In a second step, we then looked at the context of the verbs—three words to the left and three words to the right—in order to determine whether another verb occurred within this domain. All instances in which the search domain did not include another verb or included either a modal verb or a verb that did not fall within the semantic class of the fixed verb were initially categorized as “exclude.” Cases in which one of the five verbs occurred with another verb from the same semantic class within the search domain were categorized as “possible SVC”—there were 128 instances of this type. In the course of the search, certain examples led us to establish a third category, for reasons to be explained below.

### Procedure: applying the SVC criteria

For the 128 instances in the “possible SVC” category, we then closely inspected the video and applied the remaining criteria (ii) to (v) introduced in section Defining Properties of Serial Verb Constructions. This coding was done separately for the whole dataset by the two authors. Agreement between coders was 89%; the 14 cases for which they initially came to different conclusions were discussed, and an agreement was reached with respect to their inclusion or exclusion. Coding was done in the following way.

In order to determine whether the two verbs belonged to the same clause, we first applied criterion (iii), the “no linking element” criterion. Our data set did not include any examples with manual linking elements. It has to be noted, however, that—despite the existence of certain manual conjunctions (e.g., plus, or, because)—coordination and subordination are often not marked by manual signs in NGT (Van Gijn, 2004). In a next step, we therefore looked at prosodic cues, most importantly pauses and the scope of non-manual markers [criterion (ii)]^12^. Subsequently, we checked criterion (iv), argument structure properties of the utterance, and confirmed that the two verbs share arguments and that one of the verbs is not (a part of) an argument of the other verb [cf. (6)]. Following the application of these criteria, 41 instances of SVCs remained. Finally, it was also clear that none of these remaining examples exemplified an idiomatic expression [criterion (v)].

With respect to our check of prosody and argument structure, a few additional explanations are in order. All 128 examples were coded for prosody, taking into account manual and non-manual markers that have been identified in previous studies (e.g., Nespor and Sandler, 1999; Sandler, 1999). To that end, every example was viewed multiple times in slow motion. We excluded examples in which a clearly visible pause separated the two verbs and/or in which a non-manual indicated that the two verbs belong to separate prosodic constituents. In (13a), both these factors apply: roll is followed by a pause (ca. 500 ms), and a non-manual question marker accompanies the intervening index and the fixed verb go. This indicates that the two verbs belong to separate clauses, a declarative followed by an interrogative. 
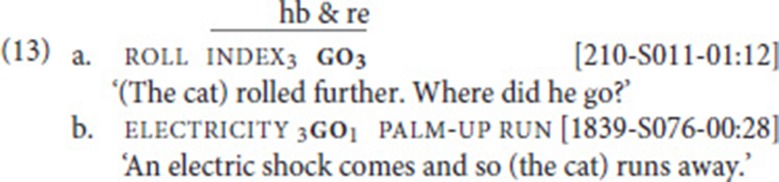


As for argument structure, we did indeed find a few examples in which the two verbs, although in close proximity to each other, did not share any arguments. One such case is exemplified in (13b) (in fact, here palm-up might function as a conjunction).

Within the “exclude” group, we encountered a couple of examples which, although not constituting SVCs according to our definition, were considered interesting from a grammaticalization perspective. As one of our goals is to offer some speculations about possible grammaticalization, we decided to create a third category “grammaticalization.” To this newly established category, we transferred: (i) ten examples involving go in which it behaves like a future marker; (ii) eight examples with give which are reminiscent of a light verb construction, that is, instances in which give combines with a noun or adjective and does not fully predicate semantically (cf. English “to give a shout”; Butt, 2010, p. 48); and (iii) 45 examples in which call appears to function as a direct speech marker. We will return to these cases, in particular those in (i) and (ii), in section Grammaticalization.

Finally, all examples extracted from the corpus were assigned a code following the scheme [video.file-signer-time.code]. Thus, code [390-S019-00:53] specifies that the example comes from video file 390^13^, is signed by signer S019, and occurs at time point 00:53. In the reported examples, Dutch glosses were translated into English, using English words that are the closest approximations of the Dutch meaning. Also note that we added subscripts to the verbs in the examples whenever it was clear for which person/object the verbs were inflected. Furthermore, for most of the Corpus NGT, no translations are provided yet. For examples including Dutch translations, we translated them into English; for the examples without translations, we offer translations that take into account the context in which the example was uttered.

## Results

An overview of the distribution of the SVCs is given in Table 1. Note that this table only includes the 41 instances that remained after applying the SVC criteria; the examples that were categorized as “grammaticalization” are not included. The table reveals that we encountered only a few SVCs per verb. This might be due to the nature of the data collection; signers engaged in more or less free conversations in a naturalistic setting. In some of the clips, signers retell a story based on elicitation materials, but these materials had not been designed to elicit specific grammatical constructions such as SVCs. In the next three subsections, we report illustrative examples of SVCs per verb. Grammatical properties of the examples, including similarities and differences between different verbs, will be discussed in section A Cross-Linguistic Perspective on Serial Verb Constructions.

**Table 1:** Number of SVCs per fixed verb in our data set, in relation to number of tokens of the four verbs.

**Verb**	***N***	**SVC**	**% of SVCs in relation to verb tokens**
go	787	21	**2.7**
give	124	4	**3.2**
take	275	4	**1.5**
call	203	12	**5.9**
	1,389	41	

### SVCs with movement verb go

**Figure 1:**
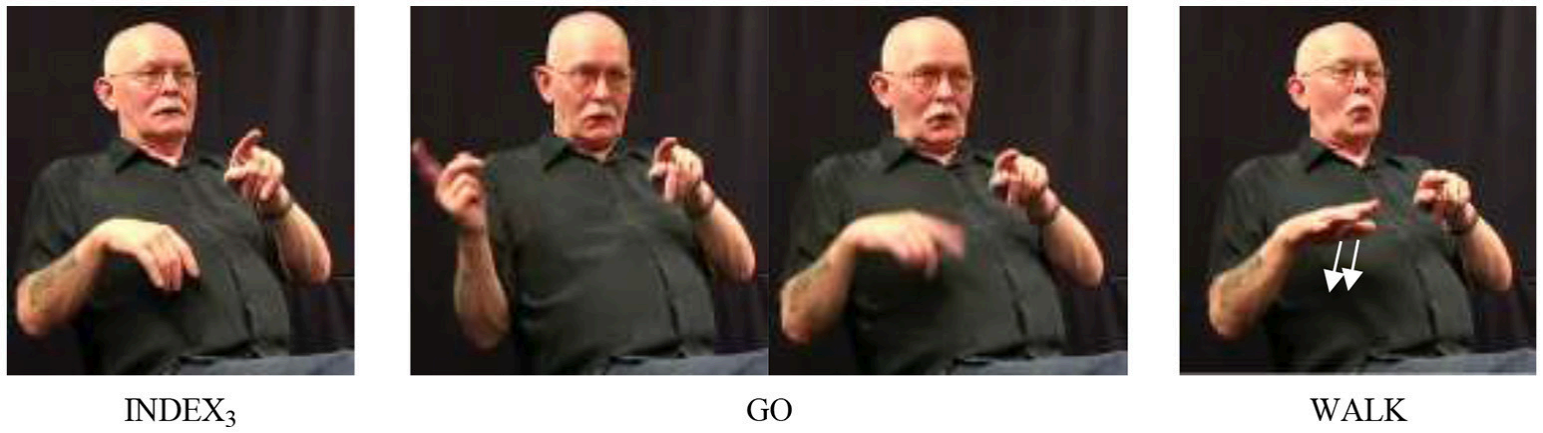
Illustration of example (14a); for the fixed verb go, the beginning and end of the movement trajectory are shown.

Our data set included a total of 787 instances of the verbs go and come (recall that we subsume come under go); these were analyzed according to the procedure outlined in section Procedure: Applying the SVC Criteria. In particular, we searched for combinations of go with other verbs of (implied) movement. This search yielded 21 cases that were analyzed as SVCs. In these examples, go combined with one of six other motion verbs, namely walk (14a) (10 instances), cycle (14b) (6 instances), hunt (2 instances), and one instance each of run, send, and swim^14^. Note that in both examples, non-manuals indicate that fixed and free verb form a prosodic unit: mouthing in (14a), pursed lips in (14b). In addition, in (14a), the index is articulated with the non-dominant hand and held while the dominant hand articulates the two verbs (weak hand spread; see Figure 1). 
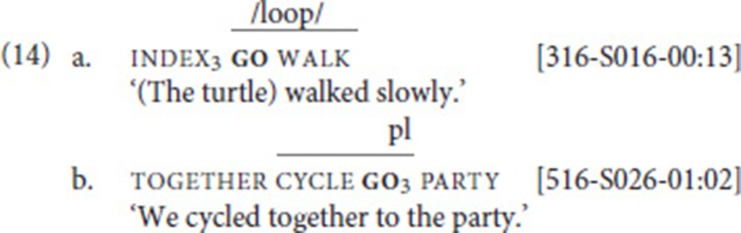


Let us briefly examine in how far our examples involving go can be compared to the “serial verbs of motion” (SVM) constructions. As described in section Serial Verbs of Motion, in SVM constructions (in ASL, LSC, and LSA), a verb expressing path combines with a manner verb; the former involves an entity classifier, while the latter involves a body part classifier. The SVCs we report above are different from SVM constructions in a number of respects. First, in our examples, the fixed verb go is a generic directional verb, which does not provide details about the moving entity or the path; that is, it never combines with an entity classifier, and it is never modified for specific path properties like “zigzag” or “upwards.” Second, the free verb does not always involve a body part classifier—only the free verbs walk, run, and swim can be analyzed as classifier predicates^15^. Third, in contrast to what has been described for ASL, LSC, and LSA, in our data, other elements may intervene between the two verbs (see Table 2 below). We thus conclude that our SVCs, while being functionally similar to SVM constructions, are structurally different. This should not be taken to imply that SVM constructions are not attested in NGT. It only means that our corpus search did not yield such constructions, as the path verb participating in the SVM construction would not be glossed as go in the Corpus NGT.

**Table 2:** Overview of attested combinations of fixed verb (go, give, take, call) and free verb, order of the fixed and free verb in the SVC, and presence and nature of intervening element (ix = index).

**Fixed verb**	**Free verb**	**N**	**Order free-fixed (*n* = 21)**	**Order fixed-free (*n* = 20)**	**Intervening element (*n* = 9)**
**go**	cycle	6	5	1	
**(*****n*** = **21)**	walk^a^	10	6	4	2 (ix, big)
	run	1	1		
	hunt	2	1	1	
	send	1	1		
	swim	1		1	1 (IX)
**give**	save	1		1	
**(*****n*** = **4)**	pay^a^	1	1		1 (ix)
	take-over	2	2		1 (ix)
**take**	replace	1		1	
**(*****n*** = **4)**	throw	1		1	
	take-with	2		2	
**call**	ask^a^	4	3	1	1 (ix)
**(*****n*** = **12)**	inform	1		1	
	sign	1		1	
	say^a^	4	1	3	3 (ix)
	request	1		1	
	thank	1		1	

a *These free verbs are also reported by Bos (1996/2016) to occur in her data set^16^*.

### SVCs with transfer verbs give and take

Bos (1996/2016) observes that the transfer verbs give and take may participate in SVCs by combining with verbs that express (concrete or abstract) transfer. We therefore searched our data set for examples in which give/take combine with another transfer verb.

**Figure 2:**
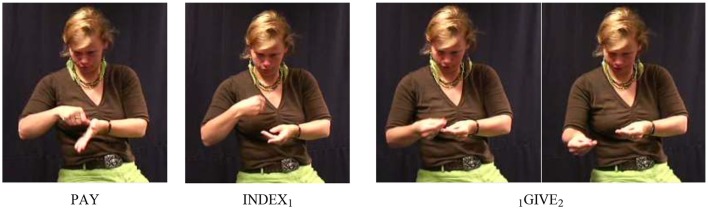
Illustration of example (15b); for the fixed verb give, the beginning and end of the movement trajectory are shown.

**Figure 3:**
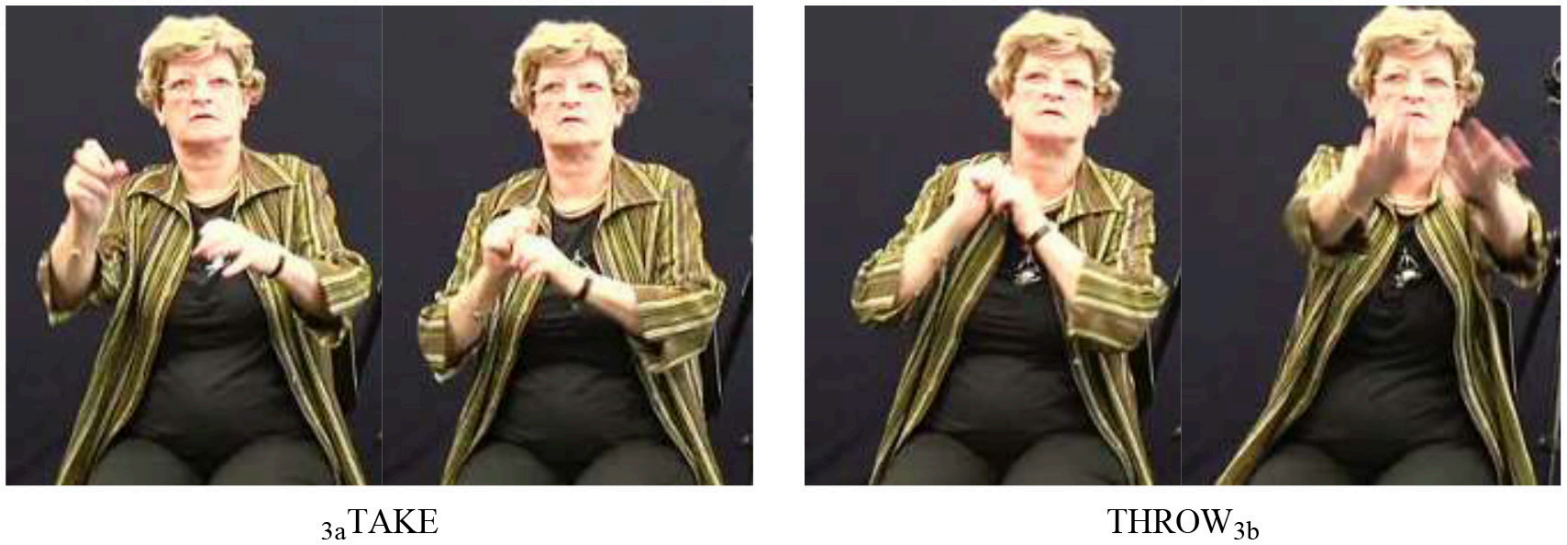
Illustration of example (16a); for both the fixed and the free verb, the beginning and end of the movement trajectory are shown.

Analysis of 124 occurrences of give yielded only four clear cases of SVCs: two instances with the free verb take-over (Dutch *overnemen*) (15a), one with pay (15b), and one with save (in the sense of “rescue”; Dutch *redden*). Clearly, in the examples involving take-over and save, the transfer semantics of the free verb are more abstract. While there is concrete transfer of an object in (15b), (15a) actually expresses the transfer of knowledge. And even for the save-case, it can be argued that it involves the abstract transfer of support from Agent to Patient (as in English “to give support”). As in (11bc), prosodic characteristics (no pause/hold) and semantics (single event) strongly suggest that we are not dealing with multi-clausal utterances—despite the intervening subject pronoun. In (15b), we also observe weak hand spread (on the non-dominant hand; see Figure 2). 
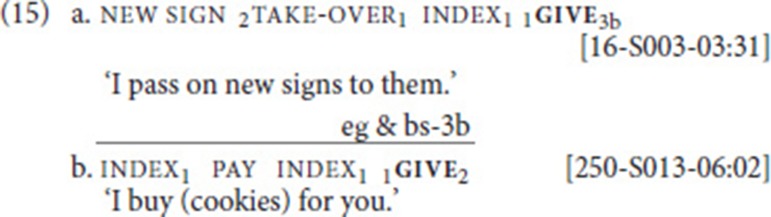


take occurred more frequently in our data set than give (275 tokens), but as with give, after applying our procedure, only four combinations that were analyzed as SVCs remained, and each one involved a different free verb, namely throw (16a), descend (16b), move, and take-with. In all SVCs involving take, the two verbs were signed with one continuous movement, so without any intervening signs (as indicated by “^∧^”; see Figure 3). 
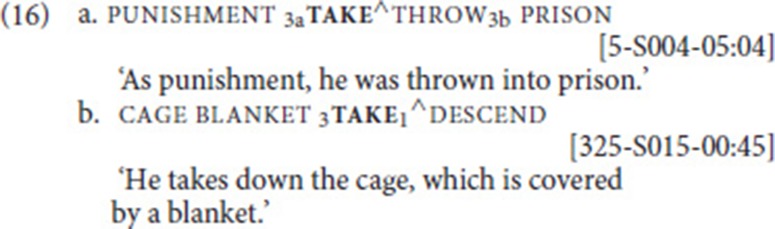


### SVCs with speech-act verb call

**Figure 4:**
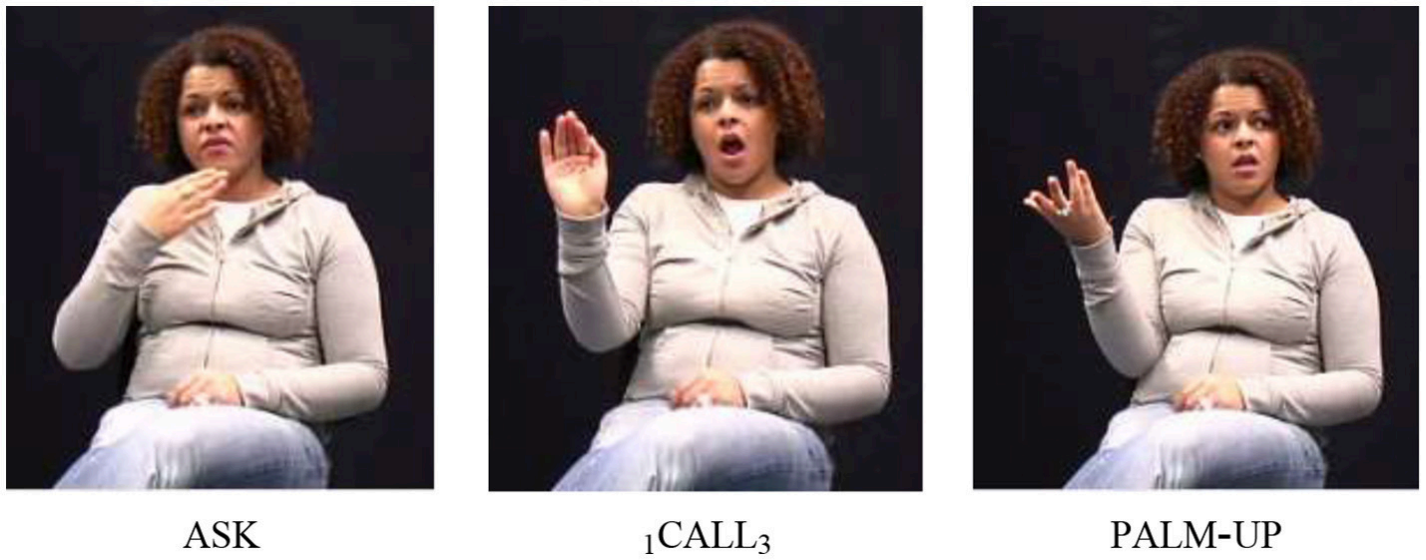
Illustration of example (17a); note that the fixed verb call does not involve path movement, only a slight hand-internal movement executed at the wrist.

Our data set included 203 instances of call. After searching for combinations with other speech act verbs and subsequently applying our procedure, we identified 12 instances of SVCs. In these examples, call (see Figure 4) combined with one of six verbs: ask (four instances) (17a), say (four instances), and one instance each of request (17b), inform, sign, and thank. Note that in example (17a), the mouthing associated with the free verb (*vragen* means “ask”) spreads over the fixed verb, thus clearly indicating that the two verbs belong together (see Figure 4). 
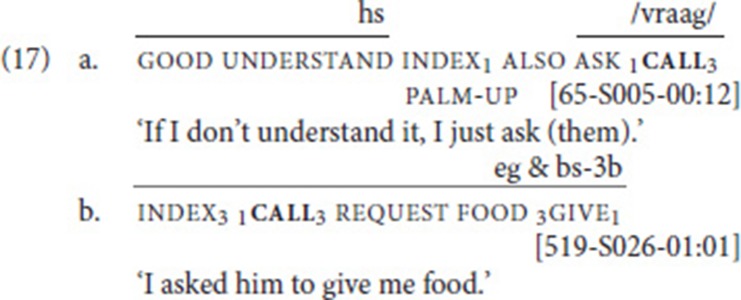


## A cross-linguistic perspective on serial verb constructions

In his insightful commentary on Bos (1996/2016), Costello (2016) offers a thorough comparison of (asymmetrical) SVCs in spoken languages and NGT. Applying ten generalizations, based on Haspelmath (2016), he re-assesses Bos' conclusion that the NGT “double verb constructions” are structurally and functionally similar to SVCs in spoken languages, and he also concludes that NGT SVCs “fit into the bigger picture.”

We shall not repeat the details of Costello's comparison here, but rather offer some additional findings concerning the structure and function of NGT SVCs, based on the corpus data. As for the generalizations, suffice it to say that Costello finds that most of them hold for the NGT data, that some cannot be applied to sign languages (e.g., shared tense value—as sign language verbs are not inflected for tense), and that two are contradicted by the NGT data. These latter two generalizations have to do with the nature of the subject: (i) if the two verbs in the SVC have different subjects, the second one is intransitive; (ii) the verbs participating in the SVC cannot have different Agents [see e.g., the Taba example in (3), where the verbs have different subjects, but the subject of the second verb is not an Agent]. Bos (1996/2016, p. 245) reports one example which does not obey these generalizations, namely index_2_ buy _1_give_2_ (“You bought it from me”), where the two verbs have different Agents, and the second verb is not intransitive. In our data, however, comparable examples did not surface, that is, the corpus data appear to be more in line with the generalizations than the elicited data.

In this section, we discuss selected structural and functional aspects of the data we extracted from the corpus, and we offer a cross-linguistic comparison of our findings, drawing on data from both spoken and sign languages, in particular the findings reported by Bos (1996/2016). In section Order of Fixed Verb and Free Verb, we start by describing the order of fixed and free verb. Subsequently, we address the nature of intervening elements (section Intervening Elements), argument structure properties (section Argument Structure Properties), and the function of SVCs (section Function of SVCs).

### Order of fixed verb and free verb

Table 2 provides an overview of all attested verb combinations in our data set, the order of the verbs within the SVC, and the occurrence of elements intervening between the free and the fixed verb.

As for the verbs participating in SVCs, we pointed out previously that the set of fixed verbs that Bos identified overlaps with verbs that are known to commonly participate as fixed verb in SVCs in spoken languages. Looking at the free verbs, we note that the set of free verbs that we identified is quite different from the free verbs reported in Bos (1996/2016). In fact, only four verbs overlap in both data sets (walk, pay, ask, say—see Table 2). Given the different data types, this discrepancy is not unexpected. Concerning the order of verbs, Bos (1996/2016) notes that in her data, the verbs in the SVC usually appear in the order free verb—fixed verb. However, she does not indicate how many of her examples contradict this pattern (and she does not provide an example of the opposite order). Our corpus data clearly indicate that the opposite order, with the fixed verb preceding the free verb, is also possible. In fact, almost 50% of our examples (20/41) show this order. Yet, different verbs show different preferences. In the following, we discuss per verb the attested combinations and the order of verbs within the SVC.

#### (i) go

As for the attested free verbs in SVCs involving go, our findings are in line with those of Bos (1996/2016), as she also observes that go combined in most of her examples with the sign walk (17/36 cases); other combinations she reports include bring, accompany, and fly. However, in her data set, no combinations with the other five verbs we found occurred. For the order of the verbs, Bos (1996/2016, p. 241) further notes that “in most double verb constructions, the fixed verb appears as the second verb,” but she does not specify how often the reverse order occurs. Our data indicate that in combinations with go, the order fixed verb—free verb is not uncommon, as it appears in 7 out 21 instances (33%)—four with walk (14a), and one each with cycle, hunt, and swim.

#### (ii) give and take

Given the small numbers for give and take, a comparison of our findings to those of Bos (1996/2016) may not be very informative. Still, we want to briefly comment on the attested combinations and the order. As for give, Bos also observes co-occurrences with pay but not with take-over or save. The most common combination in her data is with lend/borrow (21/34 cases), probably due to the elicitation procedure. Similarly, in her data, take commonly combines with lend/borrow (8/16 cases), but also with buy (7/16 cases). The combinations we found for take are not attested in her data.

Interestingly, in our data, the order is different for give and take. While give follows the free verb in three out of the four cases (the save-case being the only exception), and is thus in line with the pattern described by Bos, take always precedes the free verb. Note that Bos does not provide examples with take, but she does mention that the free verbs are initial—in contrast to what we observe. Although the small number of examples does not allow for firm conclusions, we note that the order of verbs is iconic in our examples in that it mirrors the temporal sequence of events: for take, one first has to take an entity before one can further manipulate it (e.g., by passing it on or throwing it), while in the giving event, receiving the entity (e.g., by taking it over from someone) happens before one can give it to someone (cf. Haiman, 1985). Still, we have to keep in mind that for both verbs, the reduced variation may be due to the smaller number of tokens.

#### (iii) call

In both Bos' and our data set, call most frequently co-occurred with ask and say. In Bos (1996/2016), ask accounts for 15/30 instances and say for 8/30 instances. She further reports combinations with phone and tell, but no combinations with the other three verbs we found in the corpus data. As for the order of free and fixed verb, we find that call precedes the free verb in eight out of 12 cases (17b)—again contradicting the preferred order identified by Bos (1996/2016).

Recall from section Defining Properties of Serial Verb Constructions, that iconicity has been claimed to be a factor determining the order of verbs in symmetrical SVCs (Aikhenvald, 2006, p. 28). Despite the claim that “in asymmetrical constructions the order is not necessarily iconic” (Costello, 2016, p. 255), we think that the argument can be extended to asymmetrical SVCs. In fact, studies on spoken languages suggest that within a language that features “give” and “take” SVCs, the order may also be different for the two types, as is illustrated by the Engenni examples in (18) (also see Veenstra (1996) for Saramaccan). 
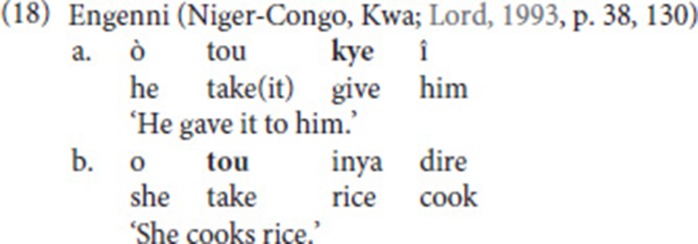


In contrast, the position of the fixed verbs go and call vis-à-vis the free verb appears to be more flexible. Once again, we believe that iconicity is at stake—or rather, the lack of iconicity in these types of SVCs. Note that in the directional case (e.g., cycle go), temporal iconicity is not involved: the movement toward a location temporally overlaps with the cycling event. Similarly, in SVCs with call, the two speech act verbs refer to the same communicative event. Given this line of reasoning, it is actually quite surprising that Bos (1996/2016) reports a fixed order also for these two verbs. Yet, it has to be noted that the two orders are not evenly distributed in our data; as is evident from Table 2, the two verbs show different preferences.

The data from the Corpus NGT, which was recorded between 2006 and 2008, thus shows more variation than the data which Heleen Bos collected in 1991. This might point into the direction of an ongoing language change. As described in section Serial Verb Constructions in NGT, NGT has traditionally been described as an SOV language. Already in 1963, Greenberg, in his famous work on language universals, noted that there is a correlation between the basic word order of a language and the order of main verbs and auxiliaries (Universal 16). In his language sample, inflected auxiliaries in SOV languages always followed the main verb (Greenberg, 1963, p. 65). This is also the most frequent order in Bos' (1996/2016) SVC data. However, a recent corpus-based study finds that SVO order is also commonly used (Oomen and Pfau, 2017). It is possible that the variation in word order goes hand in hand with variation in the positioning of the fixed verb vis-à-vis the free verb. Admittedly, however, based on our data set, such a correlation cannot be established, as only few of the SVC examples contain a direct object (in addition, subject pronouns are frequently dropped)^17^.

### Intervening elements

Another structural property that deserves attention is the nature of the intervening element. Similar to what Bos (1996/2016) described, and similar to what we know from SVCs in spoken languages (see section Defining Properties of Serial Verb Constructions), we also find that the verbs in an SVC do not have to be adjacent. Still, in our data set, an intervening element is only observed in nine out of 41 SVCs (22%); see rightmost column in Table 2. Interestingly, in eight of these cases, the intervening sign is a pronominal index (and in four out of these eight cases, a first-person pronoun). Also note that seven of the index signs are subject pronouns, and only one is an object pronoun. For instance, in (19a), a first person subject pronoun intervenes between the free and the fixed verb. In the remaining example, the intervening sign is the adjective big (19b). Note, however, that in this example, too, the intervening element, which fuses with the free verb, refers to a (previously mentioned) referent, a bear. 
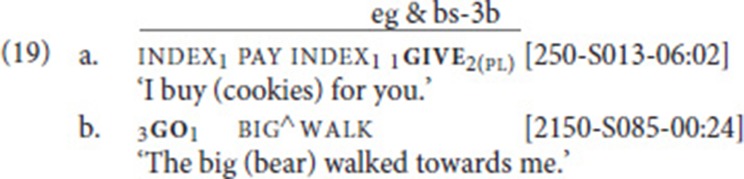


For the eight cases in which an index intervenes, we also checked whether this was more likely to occur in one of the two orders. However, no clear pattern emerged. With go, the fixed verb precedes the free verb in all three cases; with give, the free verb precedes the fixed verb in both cases; and with call, the pattern is mixed, with two examples displaying the order free–fixed and the other two the order fixed–free.

Bos (1996/2016) mentions that in about one third of her examples, an element intervenes between the two verbs, and that most frequently this element is a subject pronoun—in line with what we found. She only provides examples in which the intervener is a first-person pronoun [see (11bc) above], but mentions that nouns and adverbial signs may also intervene. We did not find such cases in our data.

Clearly, the constraint on intervening elements that we find in our data does not hold for the spoken language examples presented in section Defining Properties of Serial Verb Constructions, where full noun phrases commonly intervene between the two verbs [also see (18b)]. Yet, occasionally, SVCs in spoken languages appear to be constrained in a similar way. For instance, in one type of SVC in Degema, the two verbs, when not appearing adjacent to each other (20a), can only be separated by a mono-syllabic pronoun (20b). Rolle and Kari (2016) refer to this pattern as single-marked SVC pattern, as both verbs carry one inflectional clitic (fe = factative)^18^. 
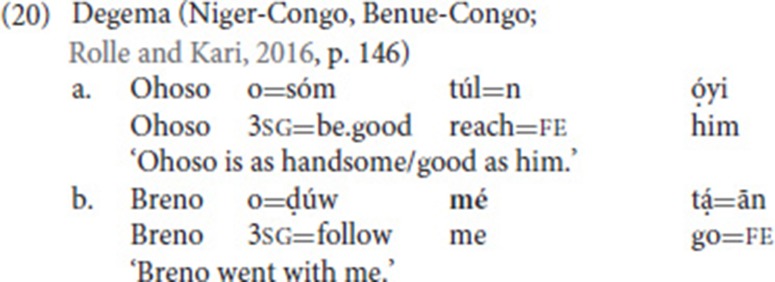


Rolle and Kari argue that the two verbs in the single-marked pattern form a verbal complex, and that the formation of this complex is sensitive to locality, where locality is measured prosodically: “If the two verbs are separated by a prosodically heavy object, the verbs are not sufficiently local for the creation of the verbal complex” (Rolle and Kari, 2016, p. 157). The clitics then attach to the edges of the verbal complex.

We propose to apply a similar line of reasoning to our NGT data. A noun (phrase) cannot intervene between the fixed and the free verb because they also form a verbal complex, and thus a prosodic constituent. Pronouns, on the other hand, are prosodically light elements that can be integrated into the complex (in fact, some of the intervening pronouns are so light that we had to check the videos a couple of times to detect them)^19^. This prosodic account is further supported by the observation that mouthings may spread from the free verb onto the fixed verb [see (14a) and (17a)]—which is indicative of prosodic integration. Yet, we only extend the prosodic account to NGT. Clearly, NGT is different from Degema in that inflectional markers do not necessarily attach to the edges of the verbal complex.

### Argument structure properties

A common characteristic of SVCs is that the two verbs share arguments. For instance, in the spoken language examples presented in section Defining Properties of Serial Verb Constructions, the second person subject is shared by the two verbs in (1); the subject as well as the direct object are shared in (2); and in (3), the object of the first verb is the subject of the second verb. We therefore also looked into the argument-sharing properties of the fixed and free verb in our data set. It turned out that the two verbs share the subject in all 41 SVCs. In addition, the object was shared between the two verbs in 15 SVCs. This pattern is illustrated in (21a), where the fixed verb call and the free verb ask share a first-person subject and a third person object (not overtly realized). In (21b), the free and the fixed verb are ditransitive, and they share the subject (first person) and the direct object (new sign); the indirect objects, however, are not shared (second person vs. third person). Our data set does not include examples in which only the object is shared, or in which the subject of one verb is the object of the other [but, as mentioned in the introduction to section A Cross-Linguistic Perspective on Serial Verb Constructions, Bos (1996/2016) reports an example of the latter type: index_2_ buy _1_give_2_ (“You bought it from me”)]. 
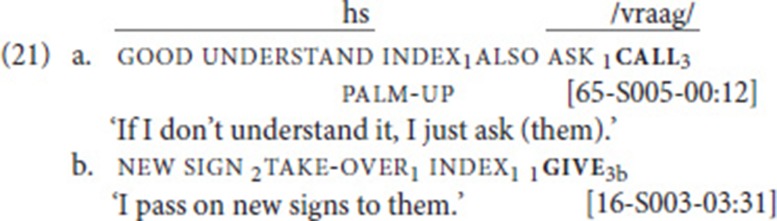


Given differences in argument structure of the participating verbs, it is not surprising that there are clear tendencies with respect to shared arguments. For instance, the fixed verb go is intransitive, and so are the free verbs it combines with; hence, we observe subject sharing in all 21 cases. The fixed verb call, on the other hand, is transitive and, for the most part, combines with other transitive speech act verbs. Consequently, in almost all cases, subject and object are shared. One case, in which call combines with the verb sign, is unclear, as sign can be used intransitively (“I sign”) and transitively (“I sign to him/her”).

### Function of SVCs

Having discussed some structural properties of our SVCs, we now turn to their function. Generally speaking, the semantic functions of the asymmetrical SVCs in our (and Bos') data set overlap with the functions described for spoken languages: (i) SVCs with go are of the directional type; (ii) SVCs with give and take are of the valency-increasing type (e.g., benefactive); (iii) SVCs with call introduce indirect or direct speech.

Beyond this interesting functional overlap, we want to address an additional issue, namely the agreement function identified by Bos. Recall from section Serial Verb Constructions in NGT, that Bos (1996/2016) finds that all free verbs in her data set are plain verbs, i.e., verbs that cannot be spatially modified to agree with their arguments. In contrast, the fixed verbs can be, and in fact are, spatially modified. Consequently, besides their semantic function, the use of SVCs is a convenient tool for expressing agreement in the context of verbs that are not capable of doing so (see section go for discussion of another element with a similar function). Hence, according to Bos, SVCs in NGT fulfill at the same time a semantic and a morphosyntactic function.

For the most part, this observation also holds for our data set. However, we also encountered some examples which are not fully in line with Bos' explanation. First, in some SVCs, there appears to be no agreement at all, as in (22a), where both verbs appear in their citation form. It should be noted, however, that occasionally an inflected form may be very similar in form to its citation form. Second, we find instances, in which agreement is marked on both the fixed and the free verb (22bc), i.e., both verbs are necessary to encode the Source-Goal relation. 
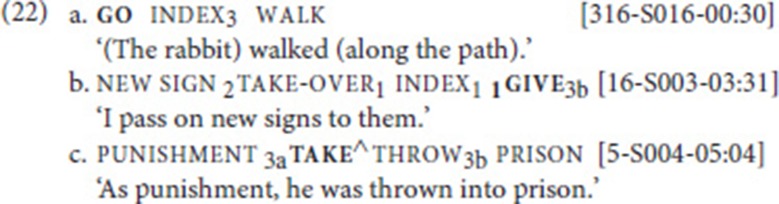


The agreement patterns are thus more diverse than what Bos describes. Obviously, this does not contradict her conclusion. On the one hand, the fact that the two data sets include different free verbs—with our set including more free verbs that can be spatially modified—may be due to different methodologies. On the other hand, the fact that SVCs are now used in a less constrained way may suggest that their semantic functions gradually become more important than their morphosyntactic function. At this point, this conclusion is only a speculation, but it is noteworthy that Bos also points out that ask, which appears as a free verb in her data, is “nowadays often used as a full agreement verb, but at the time of the recordings (1991), it was mainly used as a non-agreement verb” (Bos, 1996/2016, p. 242).

## Grammaticalization

Studies on typologically diverse spoken languages have revealed that SVCs, and asymmetrical SVCs in particular, provide a fertile ground for the grammaticalization of verbs (e.g., Lord, 1993; Veenstra, 1996; Lord et al., 2002). The fixed verb of an SVC may, for instance, develop into an adposition, aspectual marker, or complementizer. With this in mind, we now return to the third category that we established while searching the corpus data for SVCs, the “grammaticalization” category. As explained in section Procedure: Applying the SVC Criteria, some examples which did not meet our criteria for SVC, but which we considered potentially interesting from a grammaticalization perspective, were moved from the “exclude” to the “grammaticalization” category. Just as in spoken languages, grammaticalization in sign languages is characterized by loss of meaning (semantic bleaching) and often phonological erosion (for overviews, see Pfau and Steinbach, 2011; Janzen, 2012; Van Loon et al., 2014). In the following, we will discuss go and give in turn, also providing comparative examples from spoken languages and other sign languages^20^.

### GO

While analyzing the occurrences of go, we noticed that in some cases, go seemed to function as a future tense marker. In these cases, go combines with another lexical verb, but clearly does not express movement toward a location where the event expressed by the other verb took place. We found 10 examples of this type, two of which are given in (23). Of course, there were also some ambiguous combinations in which go might receive a lexical or temporal interpretation (e.g., go sleep, go meet). Interestingly, in all 10 cases, the order is go–verb, which is the opposite of the most common order in SVCs involving go (see Table 2). It is possible that this auxiliary-like use of go is influenced by Dutch, which also uses the verb “to go” (*gaan*) as a future tense marker and displays the order *gaan*—lexical verb in main clauses. 
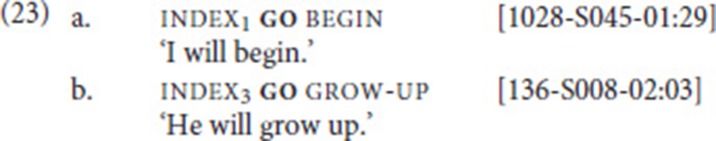


Grammaticalization of a future tense marker from the verb “to go” is common across typologically unrelated spoken languages (Bybee et al., 1991; Heine and Kuteva, 2002), but has not previously been described for NGT. However, this grammatical use has been reported for ASL. In the ASL example (24), which has been extracted from historical recordings from the 1920s, the sign glossed as future is produced twice. The second instance of future is identical to the sign go-to (which in ASL is articulated with a flat hand moving forward at waist height), while the first one is phonologically reduced: it is executed with a much shorter forward movement near the cheek. Crucially, neither of the two signs in (24) expresses physical movement (see Wilcox and Shaffer (2006, p. 227f) for epistemic use of future, in particular, the expression of future certainty). 
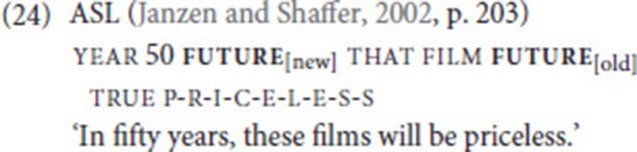


In this context, it is interesting to note that NGT features another auxiliary for which it has been claimed that it grammaticalized from the verb go: the agreement auxiliary act-on (Bos, 1994; Steinbach and Pfau, 2007). This auxiliary is void of semantics and is commonly used to express subject and object agreement whenever the lexical verb is a plain verb. In (25), for instance, act-on combines with the plain verb love to indicate a third person subject and a first-person object. Bos observes that the auxiliary is phonologically different from its lexical source: first, while go has a lax movement, the movement of the auxiliary is somewhat shorter and tense; second, act-on obligatorily combines with the Dutch mouthing /op/ (“on”). We would like to add that the handshape and orientation are also slightly different: while go is signed with an extended index finger, palm orientation to the side (see Figure 1), in act-on, the finger is usually slightly bent and the palm orientation is downwards. 
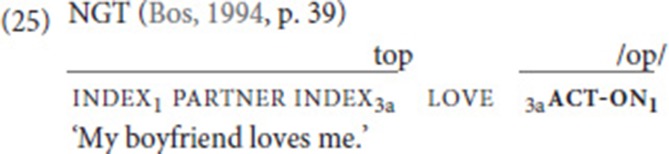


Given the observation that the expression of agreement has been shown to be an important function of NGT SVCs, Bos (1996/2016) also offers a comparison of the fixed verbs in SVCs and act-on. She concludes that act-on is clearly more auxiliary-like, as it is void of semantics and can never be used by itself in a sentence—in contrast to the fixed verbs of SVCs. This conclusion can be extended to our corpus data. We thus hypothesize that use of the movement verb go in an SVC, where it still carries semantics but already takes on the grammatical function of indicating (spatial) agreement, paved the way for its further grammaticalized use as an agreement auxiliary; in this use, the directional semantics are lost, as act-on typically realizes more abstract agreement relations (25). Semantic bleaching also characterizes the use of go as a future tense marker, but it cannot be decided whether this use constitutes a further step on the grammaticalization chain, or rather an entirely separate path. Given that ASL also features the temporal use of go, but does not employ an agreement auxiliary, we favor the latter scenario, as sketched in (26). 
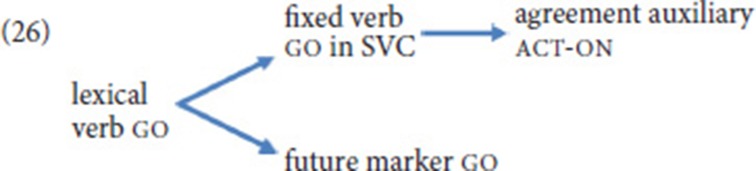


### GIVE

We now turn to the second case, the grammaticalization of give. When browsing our data set for the four fixed verbs, we encountered eight examples in which give combines with either an adjective or noun, but clearly does not express concrete transfer of an object. Such light-verb-like uses have not been previously described for NGT. Three examples are provided in (27). In (27a), give combines with the noun attention, yielding a meaning corresponding to the English “to give attention” (and Dutch *aandacht geven*). In (27b), give co-occurs with the noun blame; this use is reminiscent of the common Dutch expression *schuld geven* (lit. “to give blame/guilt”). (27c) is particularly interesting, as the combination of give with the adjective happy yields a causative meaning, similar to some causative uses of the English verb *make*. In this case, the movement trajectory of give indicates the causer and the experiencer. 
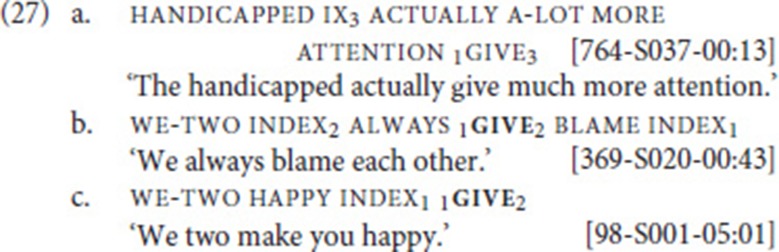


Wilcox (1998) identifies similar metaphorical extensions of the verb give in ASL, for instance, when referring to passing on knowledge or the genetic inheritance of certain physical traits. The causative use of give, on the other hand, has been described for Greek Sign Language (GSL). Sapountzaki (2005, pp. 131f) argues that the lexical verb give developed into an agreement auxiliary, which she glosses as give-aux. Crucially, unlike the NGT auxiliary in (25), give-aux does not only spell out agreement features but expresses the additional meaning of causative change of state, as illustrated in the examples in (28) (see Pfau and Steinbach (2013) for analysis)^21^. 
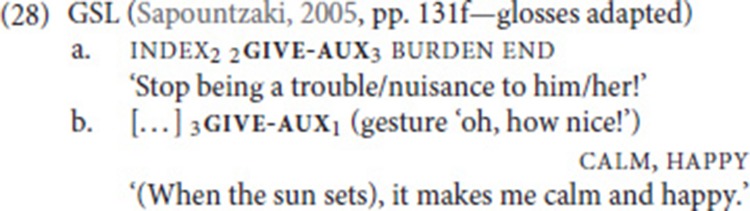


Comparable patterns of use are, of course, well-documented for spoken languages, and here, too, metaphorical extension is often the first step toward further abstraction and grammaticalization of the concept of giving. Lord et al. (2002) study the functions of “give” morphemes in SVCs (in languages of West Africa and East and Southeast Asia) and identify a number of recurrent patterns, among which Recipient/Goal, benefactive, and causative marking (see also Heine and Kuteva, 2002, pp. 149–155). The benefactive use is illustrated by the Thai example in (29), the causative use by the Akan example in (30). 
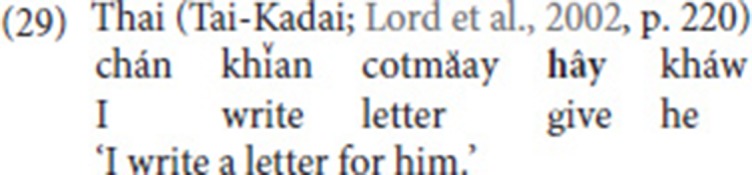
 
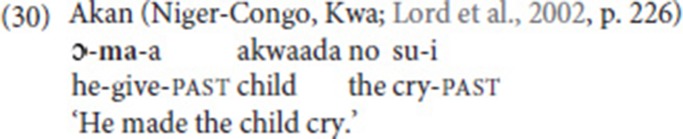


Such examples are reminiscent of the NGT examples we extracted from the corpus as well as the examples reported by Bos (1996/2016). In (11b) and (15a), the fixed verb introduces the Recipient/Goal, while in (15b), the purpose of use is benefactive marking. Finally, the causative use in (27c) is very close to the function of “give” in the Akan example in (30).

As for the grammaticalization of give, we offer the scenario in (31). When used in an SVC, give may still express concrete transfer (for instance, when combined with pay or take-over), but our data suggest that the implied transfer may also be of a more abstract nature. In this respect, the SVC overlaps with the light verb construction. In the latter, however, give combines with nouns or adjectives. The scenario in (31) further follows Heine and Kuteva (2002) and Lord et al. (2002), who demonstrate that the benefactive chain and the causative chain are separate chains. Note that give, in contrast to go, retains its agreement properties in all of its uses. 
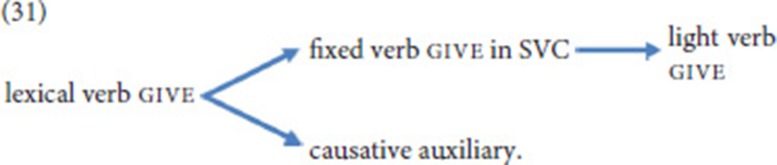


## Conclusion

The study presented here is a continuation of the seminal work by Bos (1996/2016) on SVCs in NGT (recall that her data were recorded in 1991). Analysis of 41 SVCs extracted from the Corpus NGT confirms many of the observations that Bos (1996/2016) made based on elicited data. In addition, however, the corpus data also reveal interesting differences, for instance, with respect to the order of verbs in the SVC and constraints on the intervening element. This is not unexpected, given that corpus-based studies are generally faced with more variation then might be expected based on elicited data alone. Still, the variation appears not to be random. We suggested, for instance, that temporal iconicity may have an impact on the order of the two verbs, i.e., the order is “tense-iconic” (Haspelmath, 2016, p. 309). In addition, data extracted from the corpus allowed us to offer some speculations about the grammaticalization of fixed verbs in SVCs in NGT.

It has to be acknowledged that our data set is rather small, in comparison to the data compiled by Bos by means of elicitation. In total, we found only 41 unambiguous cases of SVCs in 1,389 utterances which contained one of the four verbs go, give, take, and call. This is striking when compared to spoken languages which feature SVCs, where SVCs are usually very frequent. The low frequency of SVCs suggests that NGT has alternative strategies for encoding the information expressed in the SVCs, for instance, word order alternations or bi-clausal structures, and that such strategies are simply more commonly used in naturalistic data. It might thus be a worthwhile endeavor to complement the corpus data with grammaticality judgments in order to gain a better understanding of the grammar of SVCs in NGT. Needless to say, the study of comparable constructions in other sign languages would be a welcome addition, as it would allow for further cross-linguistic and, importantly, cross-modal generalizations.

## Author contributions

SC extracted the data from the corpus and annotated them. Both authors contributed to the theoretical background, the Methodology section, the Results section, and the section on cross-modal perspective. RP wrote the part on grammaticalization.

### Conflict of interest statement

The authors declare that the research was conducted in the absence of any commercial or financial relationships that could be construed as a potential conflict of interest.

## Footnotes

^1^It seems that Aikhenvald's approach is different in this respect, as she points out that SVCs may form lexical idioms—e.g., the combination of two verbs meaning “hit” and “split open” in Igbo to form a compound meaning “shatter” (Aikhenvald, 2006, p. 2).

^2^In our description of both agreement and classifiers, numerous complexities are neglected. Here, we only note that even applying the notions agreement and classifier to the phenomena at hand is controversial; for overviews and discussion, see e.g. Liddell (2003); Lillo-Martin and Meier (2011); Mathur and Rathmann (2012); Zwitserlood (2012), and Schembri et al. (2016).

^4^Mouthings are commonly used in NGT, be it redundantly or for the purpose of disambiguation (Schermer, 1990; Bank, 2014).

^5^While the handling classifier in (7a) refers to an object, both body part and whole entity classifiers refer to subjects (Glück and Pfau, 1998; Benedicto and Brentari, 2004).

^7^We cannot provide a comprehensive overview here, but with respect to the verbal domain, relevant studies include Bos (1990, 1993, 1994, 1998/2017), Zwitserlood (2003); Zwitserlood and Van Gijn (2006), and Oomen (2016, 2017).

^8^As for studies dedicated to SVCs in other sign languages, we are only aware of Lau (2012) and Costello's (2016) commentary on Bos (1996/2016). However, as is also evident from Costello (2016), SVCs or constructions that resemble SVCs are sometimes mentioned in the context of other construction types (e.g. motion or causation events). For the sake of cross-linguistic comparison, we provide here an example from Hong Kong Sign Language (HKSL) that is similar to the NGT example (11b). (i) HKSL (Lau, 2012, p. 174) sister egg-cake buy _0_**give**_3_ mother ‘Lit. The sister bought a birthday cake (and) gave (it) to mother.'

^9^For the sake of completeness, let us point out that the following multi-verb constructions will not be included in our study of SVCs: (i) constructions involving a “shifted referential framework,” in which the same lexical verb is signed twice but from different perspectives (Agent vs. Patient perspective), e.g. push in British Sign Language (Morgan and Woll, 2003); (ii) “verb sandwich” constructions, which are also characterized by doubling of the lexical verb but in which the second verb carries additional morphology (e.g. aspect) that is not present on the first verb (Fischer and Janis, 1990, for ASL); (iii) resultative constructions, which may involve two different lexical verbs, e.g. shake and wake-up in German Sign Language (Loos, 2017).

^10^As for other sign languages, see De Beuzeville et al. (2009) and Fenlon et al. (2018) for the spatial modification of verbs in Australian and British Sign Language, respectively, and Geraci et al. (2015) for wh-questions in Italian Sign Language.

^11^Note that call, when used in isolation, can receive various translations, such as “tell,” “inform,” and even “ask.”

^12^The nature of the data did not allow us to apply syntactic tests for mono-clausality, such as negation (actually none of our SVCs involved negation).

^13^All video files are freely available from http://www.ru.nl/corpusngt/de_filmpjes/download-filmpje-tmp/

^14^Our inclusion of the verb hunt indicates that we allow for combinations in which the free verb does not express movement per se (as e.g. walk and cycle do), but rather implies movement of the referent. In both examples with hunt, the mouthing associated with the free verb spreads onto go.

^15^In 3/10 instances, walk was not expressed by the regular sign (inverted V-hand representing two-legged entity) but by a verb that portrayed the walking of a bear or a lion, whereby the two hands represent the paws of the animal.

^16^As pointed out by a reviewer, it is likely that the number of occurrences of free verbs is related to their frequency; ask and say, for instance, are certainly more frequent than inform and request. Frequency, however, is not easily calculated in the corpus, as two-handed signs are represented on two tiers and thus counted twice.

^17^In this context, it is interesting to note that recent corpus-based work on modal verb clusters in NGT also indicates that the order of the verbs within the verb cluster may vary (Couvee and Broedelet, 2016). Traditionally the order in NGT verb clusters has been claimed to be main verb—modal verb. However, the corpus data reveal that in most clusters, the modal precedes the main verb.

^18^In addition, they describe a double-marked SVC pattern, in which both verbs carry inflectional proclitics and enclitics. This pattern appears when the two verbs are separated by a prosodically heavy (bisyllabic) direct object. We shall not discuss this pattern further, but it should be clear that we are not claiming that two distinct patterns exist in NGT, which would be distinguished by the number of inflectional markers on the participating verbs.

^19^See van der Kooij and Crasborn (2008) for the prosodic integration of pointing signs in sentence-final position in NGT.

^20^We do not discuss the possible grammaticalization of call. Across spoken languages, it is not uncommon for speech act verbs (mostly “say”) to grammaticalize into direct quotation markers (Heine and Kuteva, 2002, p. 267f) or complementizers. Bos (1996/2016) notes that call, when used in an SVC, might be on its way to grammaticalize into a direct speech marker, as it commonly directly precedes a direct speech clause (also see Van Gijn (2004, pp. 36f) for discussion). Our data set, however, does not contain examples of this type. In the few SVCs which could be interpreted as containing direct speech, the fixed verb call actually precedes the free verb [see e.g., (17b)]. Consequently, the corpus data do not allow us to argue for the grammaticalization path from fixed verb in an SVC to direct speech marker.

^21^For Catalan Sign Language, Quer and Frigola (2006; cited in Pfau and Steinbach, 2013) describe a similar causative use of give (e.g. for the meaning “it made me nervous”), and Meir et al. (2007, p. 544, footnote 10) report the use of a “periphrastic light verb construction” give fright in Israeli Sign Language to express an event of frightening.
